# What is known about the health of location-based and online web-based digital labour platform workers? A scoping review of the literature

**DOI:** 10.1186/s12889-025-23916-5

**Published:** 2025-08-02

**Authors:** Nuria Matilla-Santander, Filippa Lundh, Signild Kvart, Sherry L. Baron, Theo Bodin, Jessie Gevaert, Carin Håkansta, Julio C. Hernando, Carles Muntaner, Bertina Kreshpaj

**Affiliations:** 1 Unit of Occupational Medicine, Institute of Environmental Medicine, Karolinska Institutet, Solnavägen 4, 113 65 Stockholm, Sweden; 2 Barcelona Institute for Global Health (ISGlobal), Barcelona, Spain; 3 Universitat Pompeu Fabra (UPF), Barcelona, Spain; 4 Barry Commoner Center for Health and the Environment, Queens College, City University of New York, New York City, USA; 5 Centre for Occupational and Environmental Medicine, Stockholm Region, Stockholm, Sweden; 6 Department of Sociology, Brussels Institute for Social and Population Studies (BRISPO), Vrije Universiteit Brussel, Brussels, Belgium; 7 Research Foundation Flanders, Brussels, Belgium; 8 Department of Work Science, Karlstad Business School, Karlstad University, Karlstad, Sweden; 9 Lawrence S. Bloomberg Faculty of Nursing and Division of Social and Behavioral Sciences, Dalla Lana School of Public Health, University of Toronto, Toronto, ON Canada; 10 Section of Epidemiology, Department of Public Health, University of Copenhagen, Copenhagen, Denmark

**Keywords:** Gig work, Platformization, Occupational safety and health, Digitalization of work, Precarious employment, Algorithmic

## Abstract

**Background:**

Digital labour platforms are transforming work organization, offering new opportunities but also raising concerns about precarious conditions and health risks. Despite increasing attention to platform work, limited research has examined its direct impact on workers’ physical, mental, and social well-being.

**Objectives:**

The objective of this scoping review is to examine current empirical studies investigating the health effects of working via digital labour platforms, aiming to (i) summarize the existing evidence, (ii) pinpoint knowledge gaps, and (iii) identify areas for methodological enhancements.

**Methods:**

We search for peer-reviewed studies published until December 2024 from Web of Science and PubMed, alongside grey literature. Inclusion criteria covered papers with original data, using qualitative, quantitative, or mixed methods, resulting in 40 included studies. A pre-established theoretical framework guided result reporting, emphasizing three characteristics affecting worker health: (i) business practices, (ii) employment conditions, and (iii) work environment hazards.

**Results:**

In summary, literature shows a link between digital platform work and poor health. The current evidence, mainly focused on mental health and location-based platform workers, highlights factors contributing to poor physical and mental health, including low-quality employment conditions and psychosocial work environment hazards. Limited evidence suggests a correlation between business practices—algorithmic management and rating systems—and poor mental health. Knowledge gaps include the health impact of web-based platforms, especially medical consultation ones and location-based domestic and care services platforms, and less-explored outcomes like musculoskeletal pain and occupational injuries. Methodological limitations, such as low sample size and lack of control groups, were noted.

**Conclusions:**

This review identifies methodological improvements and knowledge gaps, guiding future research to comprehend the impact of digital platform work on health. As legislation evolves to enhance platform workers' job conditions, researching their health is crucial for offering practical recommendations and shaping evidence-based policies.

**Supplementary Information:**

The online version contains supplementary material available at 10.1186/s12889-025-23916-5.

## Introduction

Digital labour platforms are a growing feature of the modern labour market, resulting in new ways of organising work and changing how we understand and relate to labour. This change comes with positive aspects, such as lower entry barriers to work [1], but also with relevant challenges, like increased exposure to occupational health hazards among workers and an increasing share of precariously employed workers [2]. There is not a single definition of digital platform work, although it is generally understood as a matching of demand and supply of paid work, where three parties are involved: a digital labour platform, a client, and a digital platform worker [3–7]. Digital labour platforms can be classified as online web-based platforms (i.e., programming, graphic design or transcription) and location-based platforms (i.e., at a specific location such as delivering of goods, taxi- or household work) [8–10].

Estimates of the number of digital platform workers vary considerably based on degree of exposure/dependency to the job (frequency, hours and income generated through platform work) [5]. By 2022, 3% of the European working population had performed at least one hour of digital platform work in the last 12 months [11]. In the case of the U.S the estimates for those who ever provided labour via location-based platforms is of 16% in 2021 [12]. Digital platform work is rapidly expanding in the in low and medium income countries [8, 13]. In India for example, those that offer online platform services accounted for approximately 35% in 2020 [8].

Even though most digital platform workers perform tasks that are not new in the labour market, it is noteworthy that platform work has a transformative potential. Digital labour platforms have a novel and potentially disruptive role as intermediaries, shaping the status and working conditions of individuals in ways that differ from traditional employment structures [8]. This can result in ambiguity regarding the employment status of workers, as well as issues related to occupational safety and health (OSH) rights, and responsibilities [14]. Location-based and web-based platform workers differ in employment conditions, safety risks, socio-demographics, motivations, and employment trajectories. As a result, their health outcomes and the pathways to poor health are likely to differ, making it important to separate these two groups when studying the health effects of platform work.

Another important consideration when studying platform work and its association with health is understanding what distinguishes it from other forms of non-standard employment, particularly in terms of any unique risks it may pose. Some reports suggest that platform work does not introduce entirely new occupational risks; rather, the risks observed often relate to the nature of the occupation itself (e.g. traffic exposure for delivery workers or health impacts associated with self-employment) [15]. However, due to limitations in study designs, such as the lack of control groups comparing platform workers with non-platform workers performing the same job, it remains challenging to disentangle the specific contribution of platform-based work arrangements from occupation-related risks. As a result, the interaction between platform-specific risk factors and broader occupational or employment-related risks is still not fully understood.

While many studies assess the characteristics of platform work, such as business practices or the job conditions of the workers, few delve into its health implications, especially considering the distinctions between location- and web-based platform workers. Further, the interaction of platform-specific risk factors with other known occupational and employment-related risk factors remains unclear.

Given the broad and emerging nature of research on digital platform work and health, covering a wide range of outcomes, populations, and methodological approaches, we chose to conduct a scoping review. This approach is particularly suitable for mapping key concepts, types of evidence, and research gaps in a field that is still developing. It allows us to synthesize diverse findings without applying restrictive inclusion criteria, and to inform future research and policy directions. Therefore, the objective of this scoping review is to examine current empirical studies investigating the health effects of working via digital labour platforms in web-based and location-based platform workers, aiming to (i) summarize the existing evidence, (ii) pinpoint knowledge gaps, and (iii) identify areas for methodological enhancements.

## Methods

### Search strategy and eligibility criteria

The literature search included peer-reviewed studies published until December 2024 in the following electronic databases: Web of Science and PubMed. We also searched for studies in grey literature published by the ILO, EU-OSHA, Eurofound, ETUI, Fairwork, Appjobs Institute and Digital Future Society. Further, relevant studies in the references of the included studies were screened. We also screened relevant editorials and commentary papers and asked other researchers about relevant references. We also used ResearchRabbit and Litmaps to find potential papers that related to the included papers in the review. The search strategy included key words related to digital platform work, gig economy and health related outcomes (Table S1).

The eligibility criteria were: (i) papers or reports that include original data; (ii) papers or reports that explore the health of digital platform workers to any extent; (iii) studies using qualitative, quantitative, mixed or multi methods approaches, (iv) papers or reports that were written in any of the languages understood by the research team, English, Swedish, Spanish, Catalan, Italian, Norwegian, Dutch and Azerbaijani, (v) study population is based and/or includes digital platform workers. The definition used for health was the one used by the WHO: “A state of complete physical, mental, and social well-being and not merely the absence of disease or infirmity” [16]. We did not apply any specific definition for digital platform work as inclusion criteria because of the diversity of definitions used. Instead, we discussed the implications of this in the discussion.

### Study selection and data extraction

Two co-authors did a first screening of the titles and abstracts of the studies, followed by full-text reviews with data extraction done by one co-author and reviewed by another reviewer (random co-author). The study followed the PRISMA-ScR (Preferred Reporting Items for Systematic Reviews and Meta-Analyses extension for Scoping Reviews) Checklist to ensure transparent and structured reporting [17].

Information extracted from each paper was the following: year of publication, aim of the study (aimed at studying the health of platform workers or not), the design of the study (qualitative, quantitative or mixed method), the recruitment of participants (representative sample, for the case of quantitative studies), the type of platform workers studied (location-based, web-based or mixed), use of a control group (for the case of quantitative studies), type of health outcome studied, use of a validated instrument to measure the health outcome (for the case of quantitative studies), the general results related to health, the specific characteristics of platform work that affected health (classified in business practices, employment conditions and work hazards), and axes of inequality (if the studies explored results separately according to axes of inequality] [18].

To extract the information on types of digital platform work studied, we followed the ILO classification of digital labour platforms which includes online web-based platforms and location-based platforms. Web-based platforms are divided into freelance and contest-based, microtask, competitive programming and medical consultation. Location-based platforms are divided into taxi, delivery, home services, domestic work, and care services [3].

### Theoretical framework

Given the broad scope of this scoping review, covering all empirical studies that examine the health of platform workers across a wide range of potential health determinants, we identified the need for a clear and structured approach to synthesising and reporting the findings. To address this, we developed a theoretical framework prior to conducting the review. The framework was informed by the research team’s expertise, conceptual discussions, and a review of relevant existing frameworks. It was used to organise and interpret the diverse body of evidence identified through the review process.

In developing the framework, we drew on two key strands of prior work. The first included frameworks specifically addressing characteristics of platform work, such as algorithmic management [19] or job quality [20]. These models were valuable but generally focused on single aspects and did not capture the full range of factors potentially influencing health. The second strand included broader conceptual models related to the general working population, particularly those concerning precarious employment [21, 22], and occupational health and safety [23]. By integrating insights from both areas, we developed a more holistic and multidimensional framework to guide our analysis. As no existing framework covered this full range of determinants in the context of platform work, we considered this a key conceptual contribution of the study.

**Fig. 1:**
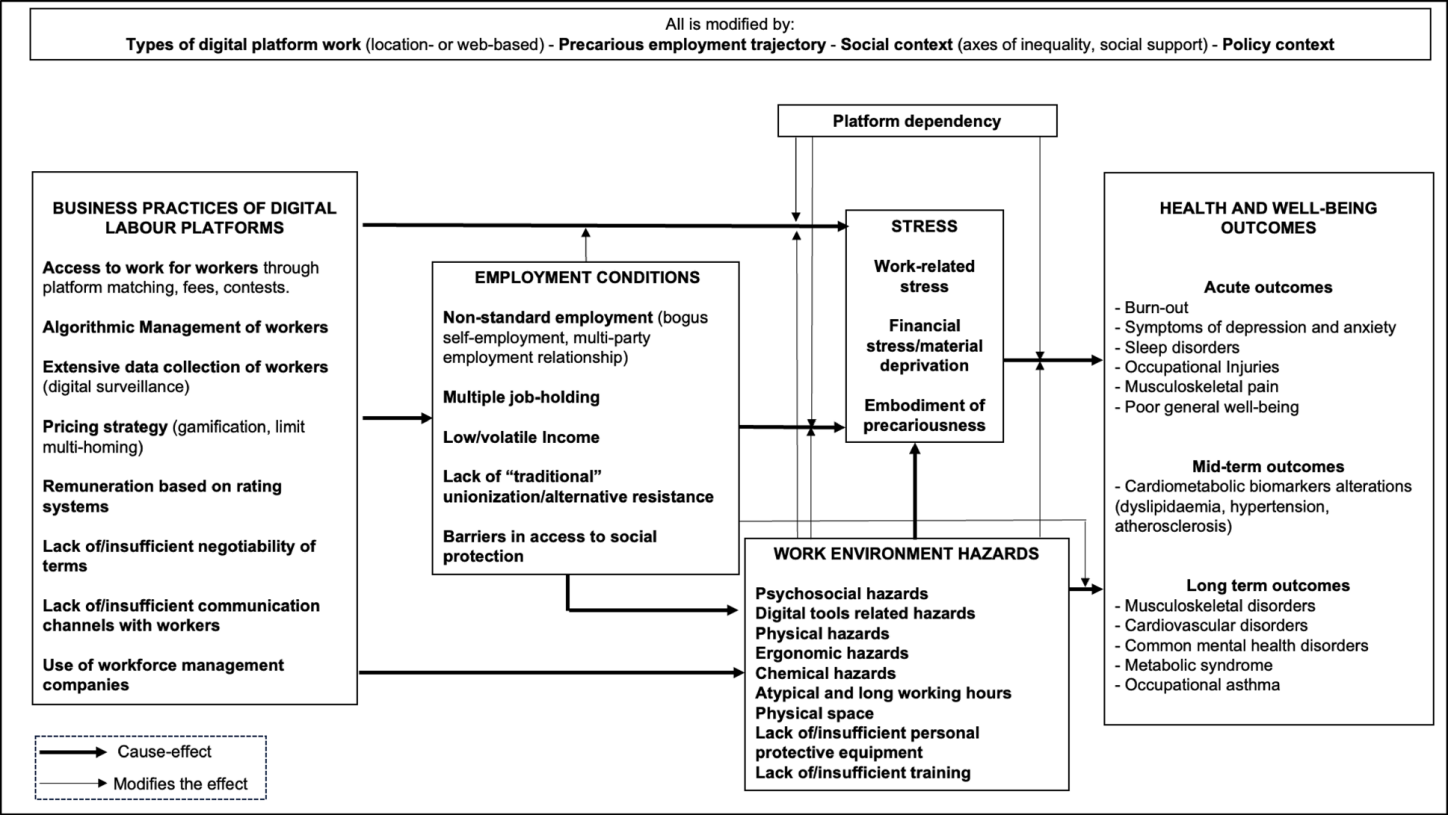
Theoretical framework showing how digital platform work may affect workers´ health and wellbeing

The framework highlights the main characteristics of platform work that we hypothesize may affect health, as illustrated in Fig. 1. The research team identified three key characteristics that may influence the health of platform workers: Business practices of digital labour platforms, Employment conditions, and Work environment hazards.

#### Business practices of digital labour platforms

These include distinct features of how the platforms connect with the workers and manage them. Platforms rely on algorithms to manage the allocation of tasks or projects, monitoring the work, and evaluating the performance of platform workers partially or fully, known as *algorithmic management* [6, 24]. The source code of the algorithms used is not transparent, but it is known that the *platforms collect extensive amounts of individual level data* of workers to feed the algorithms. This data collection relies, among other things, on the *digital tools* that workers need for working (i.e., GPS monitoring, capture of screenshots) [8]. Further, as part of algorithmic management, *remuneration is based on rating systems*. Platforms rate workers based on their performance and customer feedback, and these ratings are used to determine better or worse remuneration. According to the *rules of governance* of the platforms, this data is fully owned by the platforms, and there is a lack of access or many barriers for the workers to access it. Platforms follow a *revenue model* based on a *pricing strategy* that aims to create *network effects* (attract enough workers and customers). They do so by decreasing the prices for customers and offering economic incentives, bonuses or subscriptions to workers [8]. This is known as *gamification*, which refers to the incorporation of game elements in non-game contexts (e.g., points, badges, leaderboards, challenges) to increase engagement among workers and consumers, motivating them to accomplish tasks [25]. Additionally, they limit *multi-homing,* where workers engage with multiple platforms at the same time [26]. Furthermore, the *lack of or insufficient negotiability of terms* is a defining characteristic of how digital labour platforms operate, creating significant power asymmetries between the platform and its workers [27]. This includes engaging with the workforce through platforms' terms of service agreements, which workers must accept to gain access to the platform and begin working. Additionally, the dispute resolution services offered by these platforms often rely on unilateral mechanisms or require workers to pay for access to such services. This is also aggravated by the *lack of or inaccessible communication channels* between the worker and the platform. Furthermore, the adoption of new legislation requiring digital platforms to correctly classify workers as salaried employees has had some negative consequences. Many platforms have resorted to using *workforce management companies* to employ platform workers, particularly location-based platform workers, which perpetuates non-standard employment among platform workers [28].

#### Employment conditions

These involve *non-standard arrangements*, such as disguised self-employment. Many digital labour platforms see themselves as intermediaries connecting self-employed platform workers with clients (5). Disguised self-employment occurs when workers, despite being labelled as self-employed, experience close supervision, and their pay is routed through the platform [29]. This situation leads to various challenges for workers, including *limited access to social protection* like sickness benefits and occupational safety and health (OSH) protection [15]. Additionally, they face *low or unpredictable income* as they bear all job-related expenses. This, coupled with high competition, could prompt them to take on *multiple jobs*.

#### Work environment hazards

These are quite specific to the occupation and tasks they have to perform [30]. These might include a wide range of exposures to *psychosocial hazards* (i.e., low control, social isolation, discrimination) [31], *physical hazards* (i.e., low temperatures), *ergonomic hazards* (i.e., heavy and repetitive work), *chemical hazards* (i.e., exposure to cleaning supplies), *atypical and long working hours* (i.e., shift work or night work), *lack of or insufficient personal and protective equipment* (i.e., homologated helmet for driving) and *lack of or insufficient safety training*, *traffic-related hazards* (i.e., heavy traffic), *digital tools related hazards* (i.e., technostress, exposure to screen).

#### Impact on health

The health impact of business practices, employment conditions, and work environment hazards can be mediated through stress responses or direct effects. For example, low income can lead to poor mental health via stress, while ergonomic hazards can cause musculoskeletal pain. Health effects can be acute (e.g., sleep disorders) or long-term (e.g., musculoskeletal disorders), with prolonged exposure to platform work increasing the risks. These impacts are influenced by factors like the type of platform work (location-based or web-based), the individual's employment trajectory, and the social and policy context. Additionally, financial dependency on platform work may alter health outcomes [32]. Finally, these three characteristics can interact with each other, either exacerbating or attenuating the health impact. For example, algorithmic management (business practices) can increase job demands (work environment hazard) for delivery workers, which may negatively affect their health. At the same time, existing employment conditions in delivery work, such as low income, may amplify the harmful effects of algorithmic management through increased job demands.

## Results

**Fig. 2:**
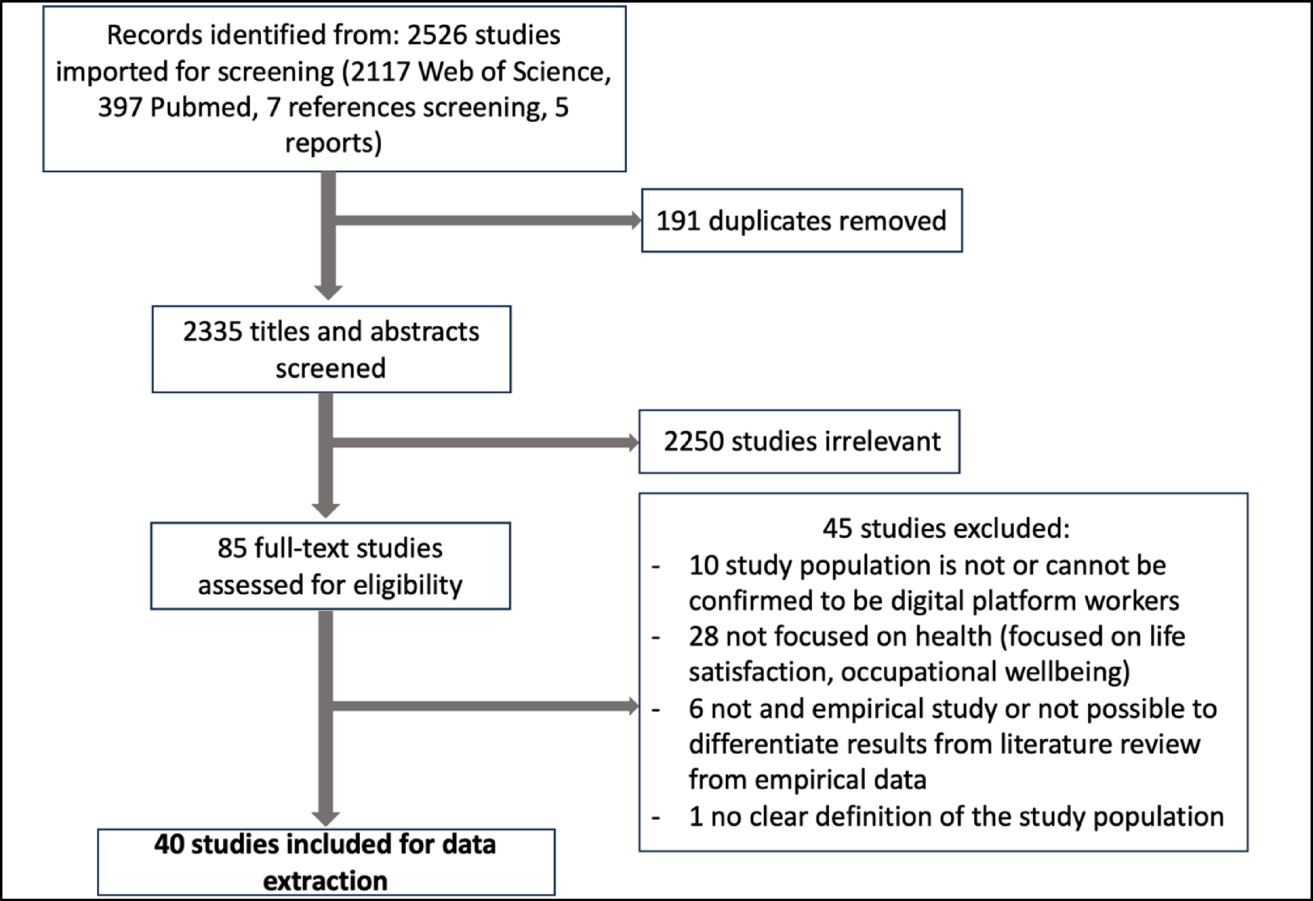
Scoping review flow diagram

The search identified 2525 studies for title and abstract screening (Fig. 2). After removing duplicates, 2334 titles and abstracts were screened, and 2250 were considered irrelevant. From these, 85 were fully text reviewed for eligibility, and 45 studies were further excluded because did not fulfil the inclusion criteria, and 40 studies were finally included for data extraction. The first included study dates from 2017.

Table 1 summarizes the characteristics of the studies in the review. Most studies focused on location-based platform workers (28 out of 40), particularly taxi and delivery workers, while web-based platform workers, such as freelancers and micro-taskers, were studied less. The studies were predominantly based in Europe and Asia and used non-representative populations. Most studies were quantitative (cross-sectional), and did not include control groups, except for a few that compared non-standard or salaried workers. Mental health was the most common health outcome studied. While the eligibility criteria allowed for studies addressing any aspect of health, most studies identified in this review focused on negative health outcomes.

**Table 1:** Characteristics of the studies included, and health outcomes studied

	N° of papers (40)
Type of platform work studied	
Only web-based	3
Only location-based	28
Both types	9
Subtypes of platform work studied	
Online web-based platforms	
Freelance	8
Microtask	5
Competitive programming	1
Contest-based	0
Location-based platforms	
Delivery	25
Taxi	20
Domestic work	7
Care services (beauty services)	2
World region of the study population (°)	
Asia	14
Africa	1
Europe	15
Latin America and the Caribbean	2
Northern America	10
Oceania	1
Study design	
Quantitative (cross-sectional)	19
Quantitative (longitudinal)	1
Qualitative	12
Mixed methods	7
Multi-methods	1
Representativeness of the sample	
Non-representative sample	34
Representative sample	6
Use of control group	
No	24
Yes	16
Health outcomes studied	
Mental health outcomes (stress, anxiety, depression, sleep problems, psychological wellbeing)	17
Musculoskeletal pain and physical strain	6
Occupational injury	5
General and somatic health	3
Infectious diseases (Covid-19)	2
Others (eyestrain, headache, weight gain, digestive issues, sedentary behavior)	4
Use of a validated instrument for measuring health outcomes	
No	8
Yes	17
Not applicable (qualitative studies)	15

(°) One study was conducted in all the regions except Oceania and one study was not possible to identify the regions

(*) N = 1 studies it was not possible to identify the use of validated instruments

One paper can measure more than one health outcome as well as subtypes of platform work

Table 2 summarizes the associations found in the studies of the three main characteristics of digital platform work: business practices, employment conditions and work environment hazards that may impact the health of platform workers.

**Table 2:** Characteristics of platform work associated or not with health outcomes by type of digital platform work

	Web-based platform work (Yes, No, No data)	Location-based platform work (Yes, No, No data)	Mixed population (Yes, No, No data)
*Business practices of digital labour platforms*			
Access to work for workers	Yes: Poor mental health: client matching through the platform [33]	No data	No data
Extensive data collection of workers	No data	No data	No data
Remuneration based on rating systems	Yes: Poor mental health [33]	Yes: Poor mental health [34]	No data
Gamification	No data	Yes: Sedentary behavior [36]:	No data
Algorithmic management	Yes: Poor mental health [33, 40]	Yes: Poor mental health [35–37, 39] Yes: Physical strain [37, 38]	Yes: Poor mental health: [40, 55] No: Poor mental health: monitoring [41]
Lack of/insufficient communication channels with workers	No data	Yes: Poor mental health: lack of direct communication with the platform [35]	No data
Lack of/insufficient negotiability of terms	Yes: Poor mental health: unilateral dispute mechanisms [33]	No data	No data
Use of workforce management companies	No data	No data	No data
*Employment conditions*			
Non-standard employment arrangements	No data	Yes: Poor mental health: self-employment [34, 42] No: Poor mental health, musculoskeletal pain and occupational injuries: no differences observed between employed and self-employed delivery workers [43]	Yes: Poor mental health: on-call and on-demand work [40]
Multiple job holding	No data	No data	No: Poor mental health: [44]
Income (low income, income volatility)	No data	Yes: Poor mental health: [8, 35–38, 44–46] Yes: Occupational injuries: income dependency [47]	No data
Lack of or difficult access to workers’ rights and protection	No data	Yes: Poor mental health: no sickness benefits coverage [45], barriers to exercise rights [36] 7/4/2025 1:22:00 PM	No data
*Working environment hazards*			
Physical hazards	No data	Yes: Poor mental health: harsh weather [45] Yes: Occupational injuries: exposure to iron [55]	No data
Psychosocial hazards	Yes: Poor general and somatic health: low job control [56], poor work-life balance due to draining energy [59] No: Poor general and somatic health: job flexibility [56] Yes: Poor mental health: job insecurity [8], poor work-life balance [8]7/4/2025 1:22:00 PM	Yes: Poor mental health: low job control [34, 37], dealing with customers [36, 45, 46, 48, 49], high demands from the platform [8, 35, 37, 50, 51], low social support from co-workers [53], respect and recognition, and justice [54], poor work-life balance due to no energy to socialize [35, 37, 48], humiliating treatment [48], personal resources [51], low workplace support [37] Yes: Poor general and somatic health: challenges—opportunities, social interactions and training [58], high workload, insufficient work, unpredictability, work pressure [58], job strain [52] Yes: Occupational injuries: high workload [47], high demands [43, 57] Yes: Musculoskeletal pain/physical strain: high work demands (workpace, workload), low work control (Task discretion) and poor work-life balance [37]7/4/2025 1:22:00 PM	Yes: Poor mental health: threats, verbal abuse, being stalked [40], social isolation [40], unpredictability of tasks [55], poor work-life balance due to no distinction between leisure and work [40] and no free time [55] No: Poor mental health: low control [41]
Traffic-related hazards	No data	Yes: Poor mental health: traffic risks [8, 34, 45], parking fines [35], vehicle stolen [35] Yes: Occupational injuries: collisions and falls [50], heavy traffic environment [60] 7/4/2025 1:22:00 PM	No data
Ergonomic hazards	Yes: Musculoskeletal pain: prolonged sitting [55]	Yes: Musculoskeletal pain: repetitive movements [36, 49], backpack [43] Yes: Poor mental health: physical strenuous work and lack of rest [40]	No data
Chemical hazards	No data	No data	No data
Atypical working hours	Yes: General and somatic health: less working hours [59] Yes: Poor mental health: atypical [8], long working hours [8]	Yes: Musculoskeletal pain and physical strain: long working hours[46, 55, 61], fatigue [physical strain]: [38, 62] Yes: Poor mental health: long working hours [8, 38, 48], waiting times [8] Yes: Sedentary behaviour: long working hours [36, 63] Eyestrain and headache: [46] Yes: Digestive issues [55]7/4/2025 1:22:00 PM	Yes: Poor mental health: irregular working hours [40] No: Poor mental health: [44]
Physical space	No data	Yes: Better mental health: beauty of the urban environment [34]	No data
Digital tools related hazards	Yes: Eyestrain: exposure to screen [55]	Yes: Occupational injuries: distractions by checking the mobile phone [62]	No data
Lack of/insufficient safety training	No data	Yes: Occupational injuries [57]:	No data
Lack of/insufficient protective equipment	No data	No data	No data

### Business practices of digital labour platforms

Regarding **business practices of digital labour platforms** only one of the studies on web-based platform workers explored how these characteristics were associated with health [33]. The interviewed web-based platform workers reported poor mental well-being due to exposure to *algorithmic management*. Additionally, the *automation of client-matching* processes, with induced workers with low levels of control, was a source of stress in how they *accessed work*.

Workers also described how remuneration based on rating systems negatively affected their mental well-being, as they perceived opacity and arbitrariness of platform reputation calculations. Additionally, *unilateral dispute resolution mechanisms*, such as abrupt introduction of changes to the work-client matching algorithm or poorly justified alterations to pricing mechanisms were highlighted as further contributors to poor mental wellbeing [33].

There were more studies focused on the health effects of business practices among onsite platform workers. *Rating systems* increased the stress levels in delivery and taxi workers [34, 35]. *Gamification*, exemplified by Uber’s surge pricing, incentivized drivers to work longer hours, and they associated this with sedentary behaviour [36]. Uber employs surge pricing, raising ride costs during high demand, with drivers earning more through a multiplier, additional surge amount, or upfront fare. The type of *platform engagement with the workforce*, in this case the lack of human contact with platform employees (i.e., lack of a phone number to communicate with the platform] was perceived as a source of stress among delivery workers [35]. *Algorithmic management* was associated with poor mental health and physical strain [37, 38]. Algorithmic management used for direction purposes (i.e., automatic allocation of tasks) was associated with stress because of having to work even being very physically tired [35] and because the app provided dispatching instructions that were distracting [36]. Algorithmic management used for evaluation purposes was also associated with stress and frustration. This was due to the monitoring of the number of refusals of passengers [36] and because of the badges being awarded according to the level of performance. These badges had implications in their access to better shifts and working hours, and therefore in their income [35]. Furthermore, taxi drivers emphasized that workers are required to disregard their psychological and physical well-being while being managed by the app [39]. Algorithmic management for monitoring and evaluation purposes increased the work pace and work demands and generated psychological and physical job strains [37]. Studies that mixed location-based and web-based platform workers also found that algorithmic management is associated with poor mental health. This has been attributed to the potential for platforms deactivating workers’ accounts, which has been associated with both stress and depression [32], as well as stress due to being evaluated in real-time and having tasks automatically allocated [40]. There was only one study that did not find an association of algorithmic management with psychological distress in a mixed sample of location-based and web-based platform workers [41].

We did not find any study exploring how the extensive data collection of workers (digital surveillance) and pricing strategies linked to limit multi-homing are linked to digital platform workers health.

### Employment conditions

None of the studies that focused only on web-based platform workers researched if employment conditions were associated with health. *Non-standard employment* was associated with poor mental health among location-based platform workers and among studies with both location-based and web-based platform workers. Self-employed delivery and taxi drivers attributed high stress levels to experiences of job insecurity and instability [34]. Taxi drivers who were self-employed but who would have preferred a salaried position were especially likely to report higher levels of anxiety [42]. Working on-call and on-demand was also reported to increase fatigue among a mixed sample of location-based and web-based platform workers [40]. One paper on delivery workers did not find differences in the odds of reporting musculoskeletal pain, occupational injuries and fatigue between salaried and self-employed delivery platform workers [43]. *Multiple job-holding* did not make any difference in stress levels among a study population of mixed location-based and web-based platform workers that worked for one or multiple platforms [44].

*Low income* was linked with poor mental health and occupational injuries among location-based platform workers and mixed study populations of web-based and location-based platform workers. Location-based digital platform workers found that insufficient payment (low income combined with extra occupational expenses) was a source of stress [8], psychological distress [45], and reduced mental well-being [36]. Moreover, financial insecurity led them to work long hours which in turn increased their health burden [46]. Income unpredictability also influenced how quickly workers had to complete tasks to earn more money and created time pressure to accept tasks as quickly as possible to avoid losing income. This was experienced as a stressor and a source of psychological strain [37]. Low income and limited compensation whereas also causes of multiple job-holding, which workers related to poor work-life balance and stress [35]. Low income, particularly in combination with income dependency on the platform was associated with increased risk of self-reported occupational injuries among food delivery workers [47]. This was explained by having to keep up with the high demands of the platform whatever the external circumstances are whether due to external factors (e.g. traffic, weather) or personal factors (e.g., physical and/or mental exhaustion).

*Lack of or difficult access to workers’ rights and protection* was associated with poor mental health in two studies about location-based platform workers. Delivery and taxi drivers declared that having no insurance coverage either in case of personal sickness or to cover their vehicles was affecting their mental wellbeing [45]. Further, taxi drivers reported not being able to exercise their right of refusing passengers because platforms imposed barriers through penalties; this was reported to be a great source of stress for them [36].

### Work environment hazards

The most frequent characteristics of digital platform work related to health in the identified literature were work environment hazards. *Psychosocial hazards* were associated with poor mental health, poor general and somatic health, as well as occupational injuries in location-based, web-based, and mixed digital platform workers. Psychosocial characteristics associated with poor mental health among location-based platform workers included low job control [34, 37], dealing with customers (emotional labour) [36, 45, 46, 48, 49], high demands from the platform [8, 35, 37, 50, 51], job strain [52], low social support from co-workers [53], lack of respect and recognition, and justice [54], humiliating treatment [48], low workplace support [37] and poor work-life balance [35, 37, 48]. One study explored personal resources (self-efficacy, hope, resilience, and optimism) and described that delivery platform workers with higher personal resources had lower chances of suffering burn-out [51]. Among web-based platform workers, it was job insecurity [8] and poor work-life balance [8] and among studies using mixed location-based and web-based populations, threats, verbal abuse, being stalked [40], social isolation [40], unpredictability of tasks [55] and poor work-life balance [40, 55]. Only one study mixing location-based and web-based platform workers did not find any association between low job control and poor mental health [41]. Low job control was associated with poor general and somatic health among web-based platform workers, while the same study did not find any association with job flexibility [56]. High workload and high demands was associated with increased risk of occupational injuries [43, 47, 57], poor general wellbeing [58] and physical strain [37] among location-based platform workers. Also, insufficient work and unpredictability of work was associated with poor general well-being among location-based platform workers [58]. Poor work-life balance was also associated with poor somatic health among micro-workers and freelancers [59].

*Traffic-related hazards* were associated with poor mental health and occupational injuries among location-based platform workers. Traffic risks [8, 34, 45], receiving parking fines while working [35], and having the vehicle stolen [35] were associated with poor mental wellbeing and stress. Also, most of the couriers in a study experienced injuries as results of a fall or collision while driving [50] and heavy traffic environments [60]. *Ergonomic hazards,* were associated with musculoskeletal pain among location-based platform workers due to repetitive movements [36, 49] and the use of backpacks [43] and among web-based platform workers due to prolonged sitting [55]. Among location-based platform workers, physically strenuous work and lack of rest were also associated with poor mental health. *Atypical and long working hours* were associated with poor mental health among web-based [8], location-based [8], and mixed digital platform workers [40]. Only one study, with a mixed sample of 12 web-based and location-based platform workers, did not find differences in the stress levels according to the number of days worked [44]. Among location-based platform workers, long working hours were also associated with musculoskeletal pain [46, 55, 61] and fatigue [62], and other health outcomes such as sedentary behaviour [36, 63], stomach issues [55], eye-strain and headache [46]. *Digital tools related risks* were described in two papers. One focused on onsite platform workers (delivery workers) and found associations to increased risk of suffering occupational injuries due to being distracted from having to constantly check their mobile phones [62]. The other was focused on web-based digital platform workers and described that the exposure to screens was associated with eye-strain [55].

Only one study was focused on *safety training* and described that this may reduce occupational injuries among delivery platform workers [57].

Based on the framework, some studies in the review explored additional factors that moderate or mediate the impact of digital platform work on health. Moderator factors included platform dependency, precarious trajectories, and social context—gender, financial precarity and loneliness-, while material deprivation was identified as a mediator. Platform dependency, the reliance on income from platform work, was linked to higher rates of occupational injuries among delivery workers [64]. A longitudinal study in the UK found that transitioning from no paid work to platform work was associated with worse mental health outcomes compared to transitioning to regular employment [65], with financial precarity and loneliness explaining the mental health disadvantages for male platform workers. A Canadian study found that platform workers experienced higher levels of loneliness compared to non-platform workers, with material deprivation not fully explaining this association, suggesting other aspects of platform work contribute to these feelings [66].

Other studies focused on describing health outcomes among platform workers. A pilot study (without a control group) reported symptoms of COVID-19 among delivery workers [67]. Two cross-sectional studies, using control groups from the general working population, compared health outcomes. One study found a higher prevalence of minor and activity-limiting injuries among platform workers [68], while the other found more musculoskeletal symptoms, general fatigue, and depressive symptoms among onsite platform workers, compared to the general population [69].

General results for each study can be found in supplementary material (Tables S2–S5).

## Discussion

### Summary of evidence

The literature indicates a clear link between digital platform work and poor health. However, the current evidence suggests that the nature of these associations vary across studies and contexts, highlighting the complexity of how digital platform work impacts health. Using a predefined framework, we identified three key characteristics of platform work affecting health: platform business practices, employment conditions, and work environment hazards.

Low-quality employment conditions (e.g., low income and non-standard arrangements) and psychosocial hazards are the most studied contributors to poor physical and mental health. Limited evidence links business practices—such as algorithmic management, rating-based remuneration, gamification, and lack of communication channels—with poor mental health, physical strain, and sedentary behavior. However, the role of employment conditions and work environment hazards in modifying these effects remains understudied.

Notable knowledge gaps include the health impacts of web-based and certain location-based platform work (e.g., domestic and care services) and underexplored outcomes like musculoskeletal pain and occupational injuries. Few longitudinal studies exist, with only one included in this review. Methodological issues, such as heterogeneous study populations, lack of control groups and small sample sizes for quantitative studies, limit current research.

### Evidence on the association between digital platform work and health

This review summarizes the literature on the association between digital platform work and health, using a framework that identifies three key factors affecting workers’ health: platform business practices, employment conditions, and work environment hazards. This framework guided the summary of the evidence.

Evidence on the health impacts of platform business practices is limited and primarily focuses on location-based or mixed-platform worker populations. Studied practices include client matching, rating-based remuneration, algorithmic management, gamification, poor communication channels, and limited negotiability of terms. These have been linked to poor mental health [33–37, 39, 40, 55], sedentary behaviour [36] and physical strain [37]. However, one study found no link between algorithmic management and psychological distress [41], likely due to mixing web- and location-based workers with differing experiences.

Employment conditions were studied only among location-based or mixed-platform worker populations, not web-based workers. Key issues included non-standard arrangements (e.g., self-employment, on-demand work), low income or income dependency, and limited access to workers’ rights and protections (e.g., lack of sickness benefits). These factors were linked to poor mental health [8, 35, 36, 44–46, 46] and occupational injuries [47]. One study found no link between multiple job holding and poor mental health, but its small sample limits reliability [44].

Work environment hazards were the most studied characteristics in both web- and location-based platform work. Psychosocial hazards, such as low job control, high demands, emotional labour, and poor work-life balance, were linked to poor mental health [8, 34–37, 45, 46, 48–51, 53–55], general health [52, 56, 58, 59], occupational injuries [43, 47, 57], and musculoskeletal pain [37]. Atypical working hours, including long, irregular, and waiting times, were associated with poor mental health [8, 38, 40, 48], poor general health [59], physical strain [38, 46, 55, 61, 62], eyestrain [46], digestive issues [55], and sedentary behaviour [36, 63]. Digital tool-related hazards, such as screen exposure and phone distractions, were tied to occupational injuries [62] and eyestrain [55]. Ergonomic hazards, including prolonged sitting and repetitive movements, contributed to poor mental health [40] and musculoskeletal pain [36, 43, 49, 55]. Other less-explored hazards, like harsh weather and traffic-related risks, were associated with occupational injuries [50, 55, 57, 60] and poor mental health [8, 34, 35, 45, 55].

### Knowledge gaps

This review identified several knowledge gaps. First, most studies on location-based platform workers focused on delivery and taxi workers, with few addressing domestic or care services. Studies that did include these workers often mixed them with delivery or taxi drivers, making it difficult to assess the health effects of these specific groups. Investigating health impacts in these less visible roles, often associated with exploitation, is important [70]. Regarding web-based platform workers, there were limited studies on their health, mainly focusing on freelancers or micro-taskers, with little attention given to competitive programming and none to medical consultation services. These two subtypes have unique business practices that could act as stressors and impact workers’ health. For example, offering work through contests could serve as a novel stressor. Additionally, the lack of studies focusing on the characteristics of web-based platforms that might affect health is problematic. This is particularly important because this type of platform work is gaining popularity, and in some countries, it is the most prevalent form of digital platform work. Furthermore, web-based platform work is global, often involving workers from low- or middle-income countries and clients from higher-income regions. Its flexibility provides opportunities for individuals with disabilities or health conditions to enter the labour market [71]. Studying the risks and opportunities of web-based platform work is crucial to protect workers and ensure sustainable and equitable access to the labour market for these individuals.

The second knowledge gap identified relates to the health impact of business practices on digital labour platforms. Only one study focused on web-based platform workers, and few addressed location-based platform workers. Examining the role of business practices in workers’ health, including differences between types of platform work, is crucial because these practices are what make platform work a novel challenge for OSH. They have the potential to exacerbate existing vulnerabilities in employment and working conditions. Moreover, the business practices of labour platforms are beginning to be adopted in other sectors where platform work was previously absent, such as health care [72], in a process known as the platformisation of work [6]. Studying the health impact of these practices is therefore also essential to understanding emerging OSH challenges that affect all workers, not just platform workers.

Next, employment conditions and their impact on the health of platform workers are also understudied, particularly among web-based platform workers, where we found no studies focused on this topic. This is especially relevant for web-based platform workers, as most are self-employed or work from anywhere in the world, making it important to study the link between their health and access to work rights and protections. Studies on location-based platform workers mostly examine issues such as low income, income dependency, or volatility, while fewer studies explore multiple jobholding or barriers to accessing workers’ rights and protections. Additionally, studies investigating the health impact of self-employment do not specifically differentiate between bogus self-employment and genuine self-employment. This distinction is critical to further understanding the issue, as it is one of the primary objectives of the new EU legislation on platform work.

Another identified knowledge gap is related to the health outcomes studied. Many of the studies aimed to study the mental health of digital platform workers—fewer focused on physical health outcomes, such as musculoskeletal pain, physical strain or occupational injuries. These outcomes are also relevant to be studied, musculoskeletal disorders rank among the most prevalent work-related health issues. In more chronic cases, they can even lead to disability and early retirement [73].

### Areas for methodological improvements

We detected several limitations in the studies that future occupational health research may consider.

First, relevant details regarding how the data were collected or characteristics of the study population were missing in some papers, such as date of data collection, or sex and age of the study participants. This hampers comparability between studies as well as the interpretation of the results in the studies.

Second, many studies lacked a clear definition of digital platform work, leading to inconsistencies in which workers were included. Some mixed platform workers with individuals using other platforms, such as for renting goods (e.g., Airbnb) or social media users, which may underestimate the health effects of platform work [8]. Additionally, some studies combined web-based and location-based platform workers or other non-standard employment types, such as disguised self-employment. This mixing makes it difficult to interpret results, as these groups differ in exposure to hazards, employment conditions, and motivations. Future studies should avoid combining different types of platform workers to better understand how specific forms of platform work impact worker health.

Most studies lacked control groups, making it difficult to isolate the health effects of digital platform work from those caused by the occupation itself. For example, occupational injuries among location-based platform workers may be linked to the nature of the tasks (e.g., exposure to traffic) rather than the platform work model. Some studies with control groups had limitations, such as combining self-employed individuals with varying employment conditions or mixing web- and location-based workers. To improve comparisons, future studies should use appropriate control groups, such as workers performing similar tasks outside of platform work or workers with varying levels of platform work exposure. This would help isolate the unique health effects of platform work.

Fourth, only one quantitative study used a longitudinal design, while the rest were cross-sectional, many of which relied on unadjusted estimates. For instance, studies comparing the health of delivery and taxi workers using platforms to those not using them may be biased due to uncontrolled factors like platform dependency, prior unemployment, or previous health status. As noted in our framework, previous precarious employment trajectories—including periods of unemployment—are important variables to consider when studying the health effects of platform work. The limited longitudinal research to date hinders understanding of how prior unemployment and other baseline factors influence health outcomes among platform workers. These studies likely didn’t adjust for confounders due to small sample sizes. Ignoring important factors, such as gender, age, or financial dependency on the platform, can lead to biased conclusions. Future studies could benefit from open-access data and data repositories on OSH in digital platform work to increase sample sizes and, in turn, improve statistical power.

Finally, another observed limitation is that most quantitative studies use non-representative samples. This is likely because platform workers are a hard-to-reach population. However, some studies manage to use representative samples from Labour Force Surveys. While such studies also face limitations, such as the underrepresentation of undocumented migrants [74], it is still crucial to advance less biased recruitment strategies to better study the health effects of platform work. Non-representative samples can lead to biased health outcomes. Future research could explore collaborations with platform companies to access their compiled data, utilize national register data, or oversample digital platform workers from the general working population.

### Limitations of the scoping review process

Due to the novelty of the topic and the emerging nature of research on digital platform work, we aimed to be as inclusive as possible of all the empirical evidence published up to this point. However, it has some limitations. It did not include a quality assessment of the studies, potentially including both high- and low-quality research, which could affect the findings. The broad research questions led to diverse study designs and outcomes, challenging data synthesis. Unlike systematic reviews, this scoping review did not conduct a meta-analysis or stringent data extraction, limiting quantitative effect estimates. These limitations should be considered when interpreting the findings.

## Conclusions

This scoping review shows an increasing body of literature associating digital labour platform work with poor health, with a primary focus on mental health among location-based workers, especially taxi and delivery drivers. We propose a framework highlighting three main characteristics affecting platform workers’ health: platform business practices, employment conditions and work environment hazards.

Using this framework, we identify critical knowledge gaps, including limited research on web-based, domestic, and care platform workers, underexplored health outcomes such as musculoskeletal disorders and occupational injuries and insufficient focus on the health impacts of business practices. Methodological improvements are also needed, such as clearer definitions of platform work, the use of appropriate control groups, longitudinal study designs, and more representative sampling strategies. These findings provide direction for future research aimed at understanding and addressing the occupational health challenges posed by digital platform work.

## Electronic supplementary material

Below is the link to the electronic supplementary material.

Supplementary Material 1.

## Data Availability

All data generated or analysed during this study are included in this published article [and its supplementary information files].

## Abbreviations

COVID-19: Coronavirus disease 2019
EU: European union
ILO: International labour organization
OSH: Occupational safety and health
PRISMA-ScR: Preferred reporting items for systematic reviews and meta-analyses extension for scoping reviews
US: United States of America
UK: United Kingdom

## References

[1] Digital platform work| Healthy Workplaces—Safe and healthy work in the digital age 2023–2025. [cited 2023 Nov 28]. Available from: https://healthy-workplaces.osha.europa.eu/en/about-topic/priority-area/digital-platform-work

[2] Muntaner C. Digital platforms, gig economy, precarious employment, and the invisible hand of social class. Int J Health Serv. 2018;48(4):597–600. 30213247 10.1177/0020731418801413

[3] Markets IL, Relations L. Digital labour platforms (Non-standard forms of employment). [cited 2023 Oct 4]. Available from: https://www.ilo.org/global/topics/non-standard-employment/crowd-work/lang--en/index.htm

[4] Platform work| European Foundation for the Improvement of Living and Working Conditions. [cited 2023 Nov 21]. Available from: https://www.eurofound.europa.eu/en/topic/platform-work#:%7E:text=workRead%20more-,Platform%20work%20uses%20an%20online%20platform%20to%20enable%20organisations%20or,connected%20thanks%20to%20an%20algorithm

[5] OECD, International Labour Organization, European Union. Handbook on Measuring Digital Platform Employment and Work. OECD; 2023 [cited 2023 Nov 21]. Available from: https://www.oecd-ilibrary.org/employment/handbook-on-measuring-digital-platform-employment-and-work_0ddcac3b-en

[6] Fernández-Macías E, Urzí Brancati C, Wright S, Pesole A. The platformisation of work. Evidence from the JRC Algorithmic Management and Platform Work survey (AMPwork). Luxemburg: European Commission; 2023. (Publications Office of the European Union). Report No.: JRC133016.

[7] Glossary| Safety and health at work EU-OSHA. [cited 2023 Nov 22]. Available from: https://osha.europa.eu/en/themes/digitalisation-work/digitalisation-glossary#glossary-P

[8] ILO. World Employment and Social Outlook 2021: The role of digital labour platforms in transforming the world of work. 2021 Feb [cited 2021 May 4]. Available from: http://www.ilo.org/global/research/global-reports/weso/2021/WCMS_771749/lang--en/index.htm

[9] European Commission. Joint Research Centre. Platform workers in Europe: evidence from the COLLEEM survey. LU: Publications Office; 2018 [cited 2022 Nov 29]. 10.2760/742789

[10] Vallas S, Schor JB. What do platforms do? Understanding the gig economy. Annu Rev Sociol. 2020;46(1):273–94.

[11] Employment statistics—digital platform workers. [cited 2024 Dec 20]. Available from: https://ec.europa.eu/eurostat/statistics-explained/index.php?title=Employment_statistics_-_digital_platform_workers

[12] Gelles-Watnick MA Colleen McClain, Michelle Faverio and Risa. The State of Gig Work in 2021. Pew Research Center: Internet, Science & Tech; 2021 [cited 2023 Dec 15]. Available from: https://www.pewresearch.org/internet/2021/12/08/the-state-of-gig-work-in-2021/

[13] Heeks R, Eskelund K, Gomez-Morantes JE, Malik F, Nicholson B. Digital Labour Platforms in the Global South: Filling or Creating Institutional Voids? Rochester: Social Science Research Network; 2020 [cited 2024 Dec 20]. Available from: https://papers.ssrn.com/abstract=3645389

[14] Commission Staff Working Document Executive Summary of the Impact Assessment Report Accompanying the document Proposal for a Directive of the European Parliament and of the Council On improving working conditions in platform work. 2021. Available from: https://eur-lex.europa.eu/legal-content/EN/TXT/?uri=celex%3A52021SC0397

[15] Digital platform work and occupational safety and health: a review| Safety and health at work EU-OSHA. [cited 2022 Dec 7]. Available from: https://osha.europa.eu/en/publications/digital-platform-work-and-occupational-safety-and-health-review

[16] Constitution of the World Health Organization. [cited 2023 Nov 28]. Available from: https://www.who.int/about/accountability/governance/constitution

[17] Tricco AC, Lillie E, Zarin W, O’Brien KK, Colquhoun H, Levac D, et al. PRISMA extension for scoping reviews (PRISMA-ScR): checklist and explanation. Ann Intern Med. 2018;169(7):467–73. 30178033 10.7326/M18-0850

[18] Marmot M. Social determinants of health inequalities. Lancet. 2005;365(9464):1099–104. 15781105 10.1016/S0140-6736(05)71146-6

[19] Vignola EF, Baron S, Abreu Plasencia E, Hussein M, Cohen N. Workers’ health under algorithmic management: emerging findings and urgent research questions. Int J Environ Res Public Health. 2023;20(2):1239. 36673989 10.3390/ijerph20021239 PMC9859016

[20] Gundert S, Leschke J. Challenges and potentials of evaluating platform work against established job-quality measures. Econ Ind Democr. 2024;45(3):696–718.

[21] Bodin T, Çağlayan Ç, Garde AH, Gnesi M, Jonsson J, Kiran S, et al. Precarious employment in occupational health—an OMEGA-NET working group position paper. Scand J Work Environ Health. 2020;46(3):321–9. 31735974 10.5271/sjweh.3860

[22] Benach J, Vives A, Amable M, Vanroelen C, Tarafa G, Muntaner C. Precarious employment: understanding an emerging social determinant of health. Annu Rev Public Health. 2014;35(1):229–53. 24641559 10.1146/annurev-publhealth-032013-182500

[23] Sorensen G, Dennerlein JT, Peters SE, Sabbath EL, Kelly EL, Wagner GR. The future of research on work, safety, health and wellbeing: a guiding conceptual framework. Soc Sci Med. 2021;1(269):113593. 10.1016/j.socscimed.2020.113593 PMC10868656 33341740

[24] Lee MK, Kusbit D, Metsky E, Dabbish L. Working with machines: the impact of algorithmic and data-driven management on human workers. In: Proceedings of the 33rd annual ACM conference on human factors in computing systems. Seoul Republic of Korea: ACM; 2015. pp. 1603–12. 10.1145/2702123.2702548

[25] Woodcock J, Johnson MR. Gamification: What it is, and how to fight it. Sociol Rev. 2018;66(3):542–58.

[26] Allon G, Cohen MC, Moon K, Sinchaisri WP. Managing Multihoming Workers in the Gig Economy [Internet]. Rochester, NY; 2023. Available from: https://papers.ssrn.com/abstract=4502968

[27] Rosenblat A, Stark L. Algorithmic labor and information asymmetries: a case study of Uber’s drivers. Int J Commun. 2016;10:27.

[28] Sherer J, Poydock M. Flexible work without exploitation. 2023. Available from: https://www.epi.org/publication/state-misclassification-of-workers/

[29] Disguised employment/Dependent self-employment. 2016 [cited 2023 Nov 30]. Available from: http://www.ilo.org/global/topics/non-standard-employment/WCMS_534833/lang--en/index.htm

[30] Lenaerts K, Waeyaert W, Gillis D, Smits I, Hauben H. Digital platform work and occupational safety and health: overview of regulation, policies, practices and research. European Agency for Safety and Health at Work.

[31] Bérastégui P. Exposure to Psychosocial risk factors in the gig economy: a systematic review. ETUI; 2021 [cited 2022 Oct 6]. Report No.: 2021.01. Available from: https://www.ssrn.com/abstract=3770016

[32] Schor JB, Attwood-Charles W, Cansoy M, Ladegaard I, Wengronowitz R. Dependence and precarity in the platform economy. Theor Soc. 2020;49(5):833–61. 10.1007/s11186-020-09408-y PMC7410973 32836676

[33] Alacovska A, Bucher E, Fieseler C. Algorithmic paranoia: gig workers’ affective experience of abusive algorithmic management. New Technol Work Employ. 2024;ntwe.12317.

[34] Apouey B, Roulet A, Solal I, Stabile M. Gig workers during the COVID-19 crisis in France: financial precarity and mental well-being. J Urban Health. 2020;97(6):776–95. 32964368 10.1007/s11524-020-00480-4 PMC7508236

[35] Mbare B. Psychosocial work environment and mental wellbeing of food delivery platform workers in Helsinki, Finland: a qualitative study. Int J Qual Stud Health Well Being. 2023;18(1):2173336. 36730307 10.1080/17482631.2023.2173336 PMC9897739

[36] Bartel E, MacEachen E, Reid-Musson E, Meyer SB, Saunders R, Bigelow P, et al. Stressful by design: exploring health risks of ride-share work. J Transp Health. 2019;14:100571.

[37] Mbare B, Perkiö M, Koivusalo M. Algorithmic management, wellbeing and platform work: understanding the psychosocial risks and experiences of food couriers in Finland. Labour Ind. 2024;7:1–26.

[38] El Bourkadi S. Uber structure’s managerial algorithmic communication and drivers’ health issues: sensemaking of work strategic resistance. Front Commun. 2023 Sep 26 [cited 2024 Dec 23];8. Available from: https://www.frontiersin.org/journals/communication/articles/10.3389/fcomm.2023.1213679/full

[39] Zhang A, Boltz A, Wang CW, Lee MK. Algorithmic management reimagined for workers and by workers: centering worker well-being in gig work. In: CHI conference on human factors in computing systems. New Orleans: ACM; 2022 [cited 2022 Nov 29]. pp. 1–20. 10.1145/3491102.3501866

[40] Kim S, Kang M, Park J. Digital industrial accidents: a case study of the mental distress of platform workers in South Korea. Int J Soc Welfare. 2022;31(3):355–67.

[41] Glavin P, Schieman S. Dependency and hardship in the gig economy: the mental health consequences of platform work. Socius. 2022;8:237802312210824.

[42] Berger T, Frey CB, Levin G, Danda SR. Uber happy? Work and well-being in the ‘Gig Economy.’ 2019.

[43] Boniardi L, Campo L, Prudenzi S, Fasano L, Natale P, Consonni D, et al. Occupational safety and health of riders working for digital food delivery platforms in the City of Milan, Italy. Work Environ Health. 2024;115(5):e2024035. 10.23749/mdl.v115i5.16278 PMC11562669 39450630

[44] Hafeez S, Gupta C, Sprajcer M. Stress and the gig economy: it’s not all shifts and giggles. Ind Health. 2022;61(2):140–50. 35249894 10.2486/indhealth.2021-0217 PMC10079501

[45] Abd Samad K, Abd Rahman NH, Ismail S, Marmaya NH. Is the well-being of gig workers in Malaysia better? The reality of pain and gain. Int Rev Appl Econ. 2023;37(4):518–31.

[46] Louzado-Feliciano P, Santiago KM, Ogunsina K, Kling HE, Murphy LA, Schaefer Solle N, et al. Characterizing the health and safety concerns of U.S. rideshare drivers: a qualitative pilot study. Workplace Health Saf. 2022;70(7):310–8. 35382630 10.1177/21650799221076873

[47] Jing Z, Yuru L, Yue Z. More reliance, more injuries: Income dependence, workload and work injury of online food-delivery platform riders. Saf Sci. 2023;167:106264.

[48] Kim MS, Oh J, Sim J, Yun BY, Yoon JH. Association between exposure to violence, job stress and depressive symptoms among gig economy workers in Korea. Ann Occup Environ Med. 2023;35:e43. 38029274 10.35371/aoem.2023.35.e43 PMC10654543

[49] Liu YL, Cheng Y, Tsai PH, Yang YC, Li YC, Cheng WJ. Psychosocial work conditions and health status of digital platform workers in Taiwan: a mixed method study. Saf Sci. 2025;182:106722.

[50] Christie N, Ward H. The health and safety risks for people who drive for work in the gig economy. J Transp Health. 2019;13:115–27.

[51] Nguyen-Phuoc DQ, Nguyen LNT, Su DN, Nguyen MH, Oviedo-Trespalacios O. Deadly meals: The influence of personal and job factors on burnout and risky riding behaviours of food delivery motorcyclists. Saf Sci. 2023;159:106007.

[52] Useche SA, Robayo S, Orozco-Fontalvo M. The hidden cost of your ‘too fast food’: stress-related factors and fatigue predict food delivery riders’ occupational crashes. Int J Occup Saf Ergon. 2024;30(3):825–34. 38853658 10.1080/10803548.2024.2356997

[53] Wu PF, Zheng R, Zhao Y, Li Y. Happy riders are all alike? Ambivalent subjective experience and mental well-being of food-delivery platform workers in China. New Technol Work Employ. 2022;37(3):425–44.

[54] Wu J, Zhou J. Basic psychological need satisfaction and well-being for gig workers: a fuzzy set QCA approach in DiDi of China. Curr Psychol. 2023;42(32):28820–32. 10.1007/s12144-022-03953-8 PMC9647244 36406847

[55] Huws U, Spencer NH, Syrdal DS, Holts K. Work in the European gig economy. Research results from the UK, Sweden, Germany, Austria, The Netherlands, Switzerland and Italy. Foundation for European Progressive Studies.

[56] Reimann M, Abendroth AK. Flexible working and its relations with work-life conflict and well-being among crowdworkers in Germany. WOR. 2023;74(2):609–20. 10.3233/WOR-210908 36278374

[57] Zheng Q, Zhan J, Feng X. Working safety and workloads of Chinese delivery riders: the role of work pressure. Int J Occup Saf Ergon. 2023;29(2):869–82. 35659214 10.1080/10803548.2022.2085915

[58] Kurian JS, Bindu MN. Navigating the gig economy: exploring challenges and motivations for the wellbeing of Gen Y and Gen Z gig workers. Cogent Psychol. 2024;11(1):2357458.

[59] Schlicher KD, Schulte J, Reimann M, Maier GW. Flexible, self-determined… and unhealthy? An empirical study on somatic health among crowdworkers. Front Psychol. 2021;12:724966. 34925133 10.3389/fpsyg.2021.724966 PMC8677419

[60] Zong Y, Tsaur SH, Dai YY. Hassles of platform-based food couriers: an Asian case study. J Transp Health. 2024;34:101743.

[61] Caban-Martinez AJ, Santiago KM, Louzado Feliciano P, Ogunsina K, Kling H, Griffin K, et al. Acute musculoskeletal pain reported among rideshare drivers in the health/safety investigation among non-standard workers in the gig economy (H.I.N.G.E.) pilot study. J Occup Environ Med. 2020;62(5):e236–9. 32149940 10.1097/JOM.0000000000001849

[62] Christie N, Ward H. Delivering hot food on motorcycles: a mixed method study of the impact of business model on rider behaviour and safety. Saf Sci. 2023;158:105991.

[63] Nilsen M, Kongsvik T. Health, safety, and well-being in platform-mediated work—a job demands and resources perspective. Saf Sci. 2023;163:106130.

[64] Laskaris Z, Hussein M, Stimpson JP, Vignola EF, Shahn Z, Cohen N, et al. A price too high: injury and assault among delivery gig workers in New York City. J Urban Health. 2024;101(3):439–50. 38683420 10.1007/s11524-024-00873-9 PMC11189866

[65] Lu Z, Wang S, Ling W, Guo Y. Gig work and mental health during the Covid-19 pandemic: a gendered examination of comparisons with regular employment and unemployment. Soc Sci Med. 2023;337:116281. 37857244 10.1016/j.socscimed.2023.116281

[66] Glavin P, Bierman A, Schieman S. Über-alienated: powerless and alone in the gig economy. Work Occup. 2021;48(4):399–431.

[67] Harris MA, Kirkham TL. COVID-19 experiences, PPE, and health concerns in Toronto, Canada bicycle delivery workers: cross-sectional pilot survey. Ann Work Expo Health. 2021;65(9):1139–44. 34212190 10.1093/annweh/wxab024

[68] Morita Y, Kandabashi K, Kajiki S, Saito H, Muto G, Tabuchi T. Relationship between occupational injury and gig work experience in Japanese workers during the COVID-19 pandemic: a cross-sectional internet survey. Ind Health. 2022;60(4):360–70. 35545553 10.2486/indhealth.2022-0012 PMC9453566

[69] Yoo H, Yang M, Song JH, Yoon JH, Lee W, Jang J, et al. Investigation of working conditions and health status in platform workers in the Republic of Korea. Saf Health Work. 2024;15(1):17–23. 38496284 10.1016/j.shaw.2024.01.002 PMC10944155

[70] Rodríguez-Modroño P, Agenjo-Calderón A, López-Igual P. Platform work in the domestic and home care sector: new mechanisms of invisibility and exploitation of women migrant workers. Gend Dev. 2022;30(3):619–35.

[71] Making the future of work inclusive of people with disabilities. 2019 [cited 2023 Dec 5]. Available from: http://www.ilo.org/global/topics/disability-and-work/WCMS_729457/lang--en/index.htm

[72] Roosevelt Institute. [cited 2025 Jan 8]. Uber for Nursing: How an AI-Powered Gig Model Is Threatening Health Care. Available from: https://rooseveltinstitute.org/publications/uber-for-nursing/

[73] Lallukka T, Kronholm E, Pekkala J, Jäppinen S, Blomgren J, Pietiläinen O, et al. Work participation trajectories among 1,098,748 Finns: reasons for premature labour market exit and the incidence of sickness absence due to mental disorders and musculoskeletal diseases. BMC Public Health. 2019;19(1):1418. 31666045 10.1186/s12889-019-7753-6 PMC6821029

[74] van Doorn N, Ferrari F, Graham M. Migration and migrant labour in the gig economy: an intervention]. Rochester: Social Science Research Network; 2020. Report No.: ID 3622589. Available from: https://papers.ssrn.com/abstract=3622589 10.1177/09500170221096581 PMC10425276 37588943

