# Supplementary material for: What is known about the health of location-based and online web-based digital labour platform workers? A scoping review of the literature
Source: BMC Public Health. 2025 Aug 2;25:2635. doi: 10.1186/s12889-025-23916-5 (PMC12317597; doi:10.1186/s12889-025-23916-5)
Supplement: Supplementary file 1 — Supplementary Material 1. [file 12889_2025_23916_MOESM1_ESM.docx]

**SUPPLEMENTARY MATERIAL**

**Table S1. Search strategy of the scoping review**

| **Pubmed**  ("platform work*"[Title/Abstract] OR "gig work*"[Title/Abstract] OR "digital platform work"[Title/Abstract] OR "gig economy"[Title/Abstract] OR "sharing economy"[Title/Abstract] OR "Uber"[Title/Abstract] OR "platform job"[Title/Abstract] OR "freelance" [Title/Abstract] OR "rideshar*" [Title/Abstract] OR "crowdwork" [Title/Abstract] ) AND (health[Title/Abstract] OR mental[Title/Abstract] OR wellbeing[Title/Abstract] OR cardio[Title/Abstract] OR musculoskeletal[Title/Abstract] OR pain[Title/Abstract] OR injury[Title/Abstract] OR accident[Title/Abstract] OR depression[Title/Abstract] OR anxiety[Title/Abstract] OR sleep[Title/Abstract] OR alcohol[Title/Abstract] OR disorder[Title/Abstract] OR disease[Title/Abstract] OR illness[Title/Abstract]) |
| --- |
| **Web of Science**  ALL=(("platform work*"OR "gig work*"OR "digital platform work"OR "gig economy"OR "sharing economy"OR "Uber"OR "platform job"OR "freelance" OR "rideshar*" OR "crowdworker" ) AND (health OR mental OR wellbeing OR cardio OR musculoskeletal OR pain OR injury OR accident OR depression OR anxiety OR sleep OR alcohol OR disorder OR disease OR illness)) |

**Table S2. Characteristics of the included studies: aim, study design and population and recruitment of the participants.**

| **Author, year** | **Aim related to health** | **Study design** | **Study population (characteristics of the platform workers)** | **Recruiment of participants** |
| --- | --- | --- | --- | --- |
| **AbdSamad 2023** | No | Qualitative, interviews | n= 11, men, mostly young 20-30, 2022, Malaysia | Non-representative. On-site recruitment |
| **Alacovska 2024** | No | Qualitative, interviews | N= 53, gender-balanced, freelancers 2019 and 2021, several countries | Non-representative (via job posts on two world remote gig economy platform and snowball sampling) |
| **Apouey, 2020** | Yes | Mixed methods | n=52, men, mostly young 18-24 (delivery) and half over 50 years old (taxi), 2020, France | Non-representative. Pooling institute |
| **Bartel, 2019** | Yes | Qualitative, interviews and focus groups | 27 platform workers, 2016-2018, Canada | Non-representative. Informational flyers, posts on online forums, snowball referrals, and through direct contact |
| **Beckman 2021** | Yes | Mixed Methods | n=100 platform drivers, male-dominated, majority 35-54 years old, 2020, US | Non-representative. Through labour unions |
| **Berger 2019** | Yes | Quantitative, cross-sectional study | N=1001 uber drivers, male-dominated, 2016-2017, UK | Non-representative (representative of Uber drivers of London) |
| **Boniardi, 2024** | Yes | Quantitative, cross-sectional study | N=240 riders, male-dominated, July-November 2022, Italy | Non-representative (on-location interviews) |
| **Caban-Martinez 2020** | Yes | Mixed methods. Cross-sectional study & interviews | n=35, male-dominated, 60% 35-54 years old, 2019, US | Non-representative. Researcher requested a ride using the digital platform. |
| **Christie 2019** | Yes | Mixed methods | N= 48 (interviews), N= 231 (online survey), male-dominated, 2018, UK. | Non-representative. Targeted advertising and posting on social media forums) |
| **Christie 2023** | Yes | Mixed methods | N= 6 (interviews, N=164 (survey), male-dominated, UK | Non-representative (quota sampling) |
| **El Bourkadi, 2023** | Yes | Qualitative, interviews | n= 50 taxi drivers, male-dominated, France | Non-representative (unions, social media, …) |
| **Glavin, 2021** | Yes | Quantitative, cross-sectional | n= 4,929 (n= 593 platform workers), 2019-2020, Canada | Representative (of Canadian workforce in general) |
| **Glavin 2022** | Yes | Quantitative, cross-sectional | N = 7.381 (n= 952 platform workers), 2020-2021, Canada | Representative (of Canadian workforce in general) |
| **Harris 2021** | Yes | Quantitative, cross-sectional study | N= 35, male-dominated, mean age 32 years, 2020, Canada | Non-representative, social media adds, online recruitment |
| **Hafeez 2023** | Yes | Quantitative, cross-sectional survey | N=49 (n=12 platform workers), 63% male, 43% in 35-44 years old - only 25% did application or digital platform-based work, year? (not reported), Australia | Non-representative, Social media recruitment |
| **Jing Z 2023** | Yes | Mixed methods, semi-structured interviews and two waves survey (30 day separation) | Quantitative part: N: 5703, 97% male, mean age: 34 years old, 2020, China). Qualitative part: N:43, males, 2020, China. | Non-representative. (survey distributed via delivery platform) |
| **Kim S 2021** | Yes | Qualitative, interviews | N=30, on-site platform workers (male dominated, mean age 40) and online platform workers (gender-balanced), 2018- 2019, South Korea | Non-representative (online advertisements and snowball sampling) |
| **Kim M-S 2023** | Yes | Quantitative, cross-sectional study | N= 521, 2021-2022, male-dominated, South Korea | Non-representative (offline and online surveys) |
| **Kurian, 2024** | yes | Quantitative, cross-sectional study | N=347, male-dominated, year? India | Non-representative (online survey) |
| **Laskaris, Z 2024** | Yes | Quantitative, cross-sectional study | N= 1650 , 2022, New York City (US) | Non-representative (online survey) |
| **Liu, 2025** | Yes | Mixed methods. Cross-sectional study & interviews | N= 33 platform workers in November and December 2020 (interviews), and 484 platform workers in December 2021 and January 2022 (survey), Taiwan | Non-representative (several purposeful recruitment techniques) |
| **Louzado-Feliciano et al., 2022** | Yes | Qualitative | N=35, male-dominated, 63% 40 years old and above, 2019, US | Non-representative (requesting services) |
| **Lu Z, 2023** | Yes | Quantitative, longitudinal study (2 waves) | N= 3381 (all, n of platform workers unknown), wave 1 (2017–2019) and wave 2 (2019–2021), UK | Representative (UK national representative) |
| **Mbare 2023** | Yes | Qualitative, interviews | N= 20, male-dominated, young (36 years old or bellow), 2020, Finland | Non-representative. On-site recruitment and social media |
| **Mbare 2024** | Yes | Qualitative, interviews | "N=30, 2020-2021, male-dominated, Finland | Non-representative |
| **Morita 2022** | Yes | Quantitative | n=591 (total of 18317 individuals), 65% men, 50% aged 20-39, 2021, Japan | Representative sample, survey panel |
| **Nguyen-Phuoc 2023** | Yes | Quantitative, cross-sectional study | n=554 , male-dominates, mean age 26 years old, 2021, Vietnam | Non-representative (face-to-face surveys) |
| **Nilsen 2023** | Yes | Qualitative | N= 32 (26 on-site and 6 online), Norway 2020 | Non-representative. Social media, face-to-face, announcing on their online community, order through the app, snowballing |
| **Reimann 2022** | Yes | Quantitative, cross-sectional study | N=470, 60% men, mean age 39, 2018-2019, Germany | Non-representative, survey posted as a gig. |
| **Schlicher 2021** | Yes | Quantitative | N=748, 57% male, mean age 37 years, YEAR?, Germany | Non-representative (though platforms) |
| **Useche, 2024** | Yes | Quantitative, cross-sectional study | N=248, male-dominated, mean age 29, 2021, Colombia | Non-representative |
| **Wang 2022** | Yes | Quantitative, cross-sectional survey | N=17722, from those N= 429 gig workers, year?, UK | Representative sample |
| **Wu PF, 2022** | Yes | Quantitative, cross-sectional study | 400 platform workers, 90%male, majority young 25-25, 2020, China. | Non-representative |
| **Wu J, 2022** | Yes | Quantitative, cross-sectional study | N= 422, 66% male, 88% between 24-39 years, 2019, China | Non-representative (representative of one ridesharing company) |
| **Yoo, H, 2024** | Yes | Quantitative, cross-sectional study | N=1000,male-dominated for drivers and couriers, female-dominated for housekeepers, 2022, South Korea | non-representative (face-to-face surveys) |
| **Zhang, 2022** | Yes | Qualitative, interview and participatory design sessions | N=24, male-dominated, mean age of 36 years, 2022, USA | Non-representative |
| **Zheng, 2023** | Yes | Quantitative, cross-sectional study | N= 9133, 2019-2020, China | Non-representative |
| **Zong, 2024** | Yes | Qualitative, interviews | N= 27, male-dominated, China, 2021-2022, Taiwan and Singapore, | Non-representative |
| **ILO 2021.** | No | Multi-methods. Quantitative, survey and qualitative, interviews and open questions | ON-SITE PLATFORM WORKERS: N= 5042 (2077 Taxi platform drivers, 2965 Delivery platform workers), 2019-2020, Argentina (only for delivery) Chile, Ghana, India, Indonesia, Kenya, Lebanon, Mexico, Morocco, Ukraine. ONLINE PLATFORM WORKERS: N= 2350 crowdworkers, 2017, 75 countries + 21 interviews. + N= 609 freelancers & 190 competitive programmers) , 2019-2020, | Non-representative (many techniques). |
| **HUWS, 2017** | No | Qualitative, interviews | 15 interviews, male-dominated, mean age 39 years old, 2016-2017, UK, Estonia (only 2 interviews from Estonia) | Non-representative (on-site recruitment, recruited through survey, trade union, facebook groups...) |

**Table S3. Characteristics of included studies: type of platform workers included, use of a control group, health outcome studied and use of a validated instrument.**

| **Author, year** | **Type of platform work** | **Control group** | **Health outcome** | **Do they use a validated intrument for measuring the outcome? (Only applicable for quanti/mixed-methods)** |
| --- | --- | --- | --- | --- |
| **AbdSamad 2023** | On-site platform work, delivery and taxi | None | MENTAL. Wellbeing (being worried about) | N/A |
| **Alacovska 2024** | Online platform work, creative freelancers, in particular graphic designers, illustrators, and visual artists | none | stress, poor mental wellbeing | n/a |
| **Apouey, 2020** | On-site platform work, delivery and taxi | Yes: other precarious workers and platform workers (domestic/freelancers) | MENTAL. Anxiety, stress, and psychological wellbeing. | No |
| **Bartel, 2019** | On-site platform work, taxi | Yes, taxi drivers (traditional). But not clearly distinguished in the results because they were not part of the focus groups. | physical (musculoskeletal pain, sedentary behaviour) and mental health | n/A |
| **Beckman 2021** | On-site platform work, taxi | None | COVID‐19 infection, stress | Yes, Perceived Stress Scale-14 (PSS-14) |
| **Berger 2019** | On-site platform work, taxi | Yes, salaried and self-employed workers | Emotional wellbeing (happiness and anxiety) | No |
| **Boniardi, 2024** | Onsite platform work, delivery | None | Occupational injury, musculoskeletal pain and fatigue | No |
| **Caban-Martinez 2020** | On-site platform work, taxi | None | Muscle and joint pain | Yes, Nordic questionnaire |
| **Christie 2019** | Onsite platform work, delivery | None | Fatigue and injuries | Not applicable |
| **Christie 2023** | Onsite platform work, delivery | Yes, employed delivery workers | Fatigue and injuries | Unknown |
| **El Bourkadi, 2023** | On-site platform work, taxi | None | stress, poor mental wellbeing, physical strain | Not applicable |
| **Glavin, 2021** | On-site (taxi) & online (crowdwork) | Yes, permanent wage work, temporary wage work, independent contractor and business owner | Loneliness | "Yes, adapted from a validated three-item scale |
| **Glavin 2022** | On-site (taxi, delivery, domestic) & online (freelancers, microworkers) | Yes, permanent wage work, temporary wage work, and self-employed | Psychological distress | Yes, validated instrument (Kessler) |
| **Harris 2021** | On-site: delivery | None | COVID-19 infection | No |
| **Hafeez 2023** | On-site (ride sharing, food delivery, handyman, cleaning, retail) and online (freelancing). | Yes, other gig workers (independent contractors, temporary workers, on-call workers, supplementing with gigwork, workers that work via text/phone call) | Stress | Yes, Perceived Stress Scale-14 (PSS-14) |
| **Jing Z 2023** | On-site: Food-delivery | No | Self-reported work injury | No |
| **Kim S 2021** | On-site (food delivery, taxi) and online (freelance, content creation, accomodation) | None | mental distress (stress, fatigue, anxiety) | N/A |
| **Kim M-S 2023** | On-site platform work, replacement drivers, housekeepers, and food delivery drivers. | Yes, general workers | mental health (depression) | Yes, Patient Health Questionnaire (PHQ-9) |
| **Kurian, 2024** | On-site platform work, delivery workers, domestic work, beauty services and taxi drivers. | None | well-being | Yes, NIOSH worker well-being questionnaire (WellBQ) |
| **Laskaris, Z 2024** | On-site platform work, delivery | Yes, platform dependence (main job vs., side job) | occupational injury | No |
| **Liu, 2025** | On-site platform work, delivery, taxi, personal care, home cleaning. | Yes, general workers | self-rated health, burnout, poor mental health, and work-related injuries | Yes, Copenhagen Burnout Inventory, 5-item brief symptom rating scale (BSRS-5), |
| **Louzado-Feliciano et al., 2022** | On-site platform work, taxi | None | Physical (eye-strain, headache) and mental health | N/A |
| **Lu Z, 2023** | On-site (taxi, food delivery, couriers, other manual tasks), and online platform work (freelance) | Yes, regular employed and unemployed | Mental health | "Yes, 12-Item Short Form Health |
| **Mbare 2023** | On-site platform work, delivery | No | Mental wellbeing | N/A |
| **Mbare 2024** | On-site platform work, delivery | no | exhaustion, physical strain, psychological stress | Not applicable |
| **Morita 2022** | Onsite and online platform work | Yes, workers with no gig work experience in the last year | Occupational injury | No |
| **Nguyen-Phuoc 2023** | Onsite, delivery | none | Burn-out | Yes |
| **Nilsen 2023** | On-site (delivery) and online (freelance) platform work | No | Injuries and physical health (sedentary behaviour) | N/A |
| **Reimann 2022** | Online platform work, microworkers, crowdworkers. | No | Somatic health (stomach or bowel problems, back pain, pain in arms/legs, headaches, chest pain, dizziness, feeling tired, trouble sleeping) | Yes, Somatic Symptoms Scale (SSS-8) |
| **Schlicher 2021** | online platform work, microworkers, freelancers | Yes, regularly employed personnel | Somatic health (stomach or bowel problems, back pain, pain in arms/legs, headaches, chest pain, dizziness, feeling tired, trouble sleeping) | Yes, Somatic Symptoms Scale (SSS-8) |
| **Useche, 2024** | Onsite platform work, delivery | no | Psychological distress, fatigue, crashes | "Yes, GHQ‐12 and the need for recovery |
| **Wang 2022** | Onsite and online platform work | Yes. Workers in full-time, part-time, unemployment | Mental health | Yes, GHQ‐12 |
| **Wu PF, 2022** | Onsite, delivery | No | Mental health | Yes, GHQ‐12 |
| **Wu J, 2022** | On-site platform work, taxi | None | Psychological Well-being | Yes, EWB |
| **Yoo, H, 2024** | On-site, delivery, taxi, housekeepers | Yes, general workers | Preseentism, mental health (depression, sleep disturbance, anxiety), musculoskeletal pain (back pain, upper limb pain, lower limb pain), headache/ eye fatigue | Yes, Korean version of the Patient Health Questionnaire -9 (PHQ-9) for depression, Korean version of the Insomnia Severity Index forsleep disturbancess, |
| **Zhang, 2022** | On-site platform work, taxi | None | Psychological & Physical well-being | N/A |
| **Zheng, 2023** | On-site platform work, delivery | None | Occupational injury | No |
| **Zong, 2024** | Onsite, delivery | None | Physical health (digestive issues, musculoskeletal pain), occupational injuries | N/A |
| **ILO 2021.** | On-site (taxi, delivery) and online (freelancers, microworkers, competitive programming) platform work | Yes, for on-site workers: taxi drivers and delivery workers not working through platforms. | Self-reported general health and stress | Not possible to know |
| **HUWS, 2017** | Online (crowdwork), onsite (delivery workers, domestic, taxi) | No | Musculoskeletal pain, eye-strain, occupational injury, stress, depression | N/A |

**Table S4. Characteristics of the included studies: whether they explore business, employment or work environment characteristics related to health outcomes.**

| **Author, year** | **Do they explore if business characteristics are related to health outcomes?** | **Do they explore if employment characteristics are related to health outcomes?** | **Do they explore if working environment are related to the health outcomes?** |
| --- | --- | --- | --- |
| **AbdSamad 2023** | No | Factors related to poor mental wellbeing. **WORKERS RIGHTS AND PROTECTION.** Osh and sickness benefits coverage**:** No insurance coverage in case of sickness or for their working vehicle. **INCOME**: less orders = less income | Factors related to poor mental wellbeing. **PHYSICAL:** Harsh weather **PSYCHOSOCIAL:** bad customer's attitudes, **TRAFFIC RISKS**: traffic related risks. |
| **Alacovska 2024** | ALGORITHMIC MANAGEMENT (management through platform) + ACCESS TO WORK FOR WORKERS (client‐matching is automated) + RATING SYSTEMS IMPACT ON REMUNERATION (The opacity, arbitrariness and ‘caprice’ with which platform ‘reputation’ is algorithmically calculated) + UNILATERAL RESOLUTION MECHANISMS (brupt introduction of changes to the work‐client matching algorithm, for example, or poorly justified alterations of the pricing mechanisms = poor mental wellbeing | X | X |
| **Apouey, 2020** | **RATING SYSTEMS**. Increase stress. | **NON-STANDARD EMPLOYMENT. Self-employment =** job insecurity/instability = Higher stress. | **PSYCHOSOCIAL.** Autonomy and responsibility = higher wellbeing. **URBAN ENVIRONMENT.** Beauty of the environment = higher wellbeing. **TRAFFIC-RELATED HAZARDS**. Density of urban traffic = higher stress |
| **Bartel, 2019** | **GAMIFICATION**. Uber's surge pricing = long working hours = sedentary behaviour. **ALGORITHMIC MANAGEMENT**: directions provided by the app are distracting = Stressful, monitoring of n°of refusals of passengers = stressful. **RATING SYSTEMS**. Customer-ratings = stressful. | **WORKPLACE RIGHTS**. Barriers to exercise their right to refuse passengers because of penalties by the platforms = stressful. **INCOME INSECURITY =** lack of mental well-being | **LONG WORKING HOURS =** sedentary behavior, **PHYSICAL:** repetitive movements**=** musculoskeletal pain, **PSYCHOSOCIA**L: dealing with customers = stressful |
| **Beckman 2021** | x | x | **PSYCHOSOCIAL HAZARDS.** More job satisfaction = less stress. **WORKING HOURS**. There is a trend (not statistically significant) on decrease of working hours |
| **Berger 2019** | x | **NON-STANDARD EMPLOYMENT:** drivers that are self-employed and that would like to work as salaried workers report higher levels of anxiety. | **LONG WORK HOURS**: there is no evidence of a direct link between the mean hours spent logged into the Uber app per week and anxiety. |
| **Boniardi, 2024** | x | Employment contract. No differences between employed and self-employed delivery workers in terms of odds of fatigue, musculoskeletal pain and accidents. | ERGONOMIC. Use of backpack for riders increases the odds for backpain and fatigue. PSYCHOSOCIAL. More number of deliveries (work demands) = higher odds of accidents |
| **Caban-Martinez 2020** | x | x | **WORKING HOURS**: full-time job as platform worker and more hours/week in rideshare= higher % of musculoskeletal pain |
| **Christie 2019** | x | x | **PSYCHOSOCIAL HAZARDS**. High demands by the platform = higher fatigue. **TRAFFIC RELATED RISKS**. Most of the courirers experienced injuries as a results of a fall or collision. |
| **Christie 2023** | x | x | DIGITAL TOOLS RELATED RISKS. Checking the mobile phone = distraction = higher chance of injuries. LONG WORKING HOURS: fatigue |
| **El Bourkadi, 2023** | ALGORITHMIC MANAGEMENT. Constant surveillance, performance evaluations = stress. | INCOME. Unpredictability of earnings = stress | LONG WORKING HOURS. physical strain and poor mental well-being |
| **Glavin, 2021** | x | x | x |
| **Glavin 2022** | **ALGORITHMIC MANAGEMENT.** Mixed platform workers. Platform monitoring= No association with psychological distress. |  | **PSYCHOSOCIAL HAZARDS**. No differences of stress due to low control between platform and non-platform. |
| **Harris 2021** | x | x | x |
| **Hafeez 2023** | No | **INCOME**. Low income = More stress. . **MULTIPLE JOB HOLDING**. No stress difference between one or multiple employers/platforms. | **WORKING HOURS**. **Days worked.** Scores on PSS-14 were similar regardless of number of days worked. |
| **Jing Z 2023** | No | **INCOME.** Higher income dependency = more work injury. | **PSYCHOSOCIAL HAZARDS**. Workload mediates income dependency and work injury. Higher income dependency = higher workload = higher injuries |
| **Kim S 2021** | **ALGORITHMIC MANAGEMENT**. Performance rating system = being evaluated in real-time = stress AND Automated allocation of activities = stress . This is observed to be higher among onsite platform workers compared to online. | **NON-STANDARD EMPLOYMENT**. Working on-call and on-demand = fatigue | **WORK-LIFE BALANCE.** No clear distinction between **labour and leisure** = fatigue AND **irregular working hours** = not being able to meet people = feelings of loneliness/isolation = mental distress. **ERGONOMIC HAZARDS**. Physical strenous work (due to lack of rest in delivery workers = lack of rest = fatigue). **PSYCHOSOCIAL HAZARDS** = disclosure of private information in workers profiles = **threats and being stalked** = anxiety AND online customer's feedback = **verbal abuse** = mental distress AND **social isolation** = mental distress |
| **Kim M-S 2023** | x | x | WORKING HOURS. To work more than 40 hours per week = higher depression compared to general workers. PSYCHOSOCIAL HAZARDS. Exposure to violence and job stress = higher depression compared to general workers. Humiliating treatment, emotional labour = depression WORK-LIFE BALANCE. Poor work-life balance = depression |
| **Kurian, 2024** | x | x | PSYCHOSOCIAL HAZARDS. Challenges (measuring opportunities, social interactions and training) = poor wellbeing AND Occupational stress (measuring workload, insufficient work, unpredictability, work pressure, work environment) = poor wellbeing |
| **Laskaris, Z 2024** | x | x | x |
| **Liu, 2025** | X | X | ERGONOMIC HAZARDS. General musculoskeletal problems due to repetitive movements. PSYCHOSOCIAL HAZARDS. Depression and anxiety due to unfavorable customer attitudes. Emotional distress induced by interactions with the families of care recipients. |
| **Louzado-Feliciano et al., 2022** | x | **INCOME.** Low income + extra occcupational expenses = financial insecurity = stress = long-working hours = health burden | **WORKING HOURS.** Long working hours = prolonged time being sit = neck, back and leg pain & Long working hours = eye strain & headache. **PSYCHOSOCIAL HAZARDS**. Poor passenger behaviour/violence/disrespectful = hazard for their health = worry |
| **Lu Z, 2023** | x | x | x |
| **Mbare 2023** | **ALGORITHMIC MANAGEMENT**. **Evaluation-purposes:** Badges beign awarded for performance = implications in working hours/access to shifts = implications in income = **stress & frustration**. **Direction-purposes:** Automatic allocation of tasks = having to work when being very physicial tired = **stress. ENGAGEMENT WITH WORKFORCE.** Lack of direct phone number/communication with platform = **stress** | **INCOME.** Low income = multiple job-holding = tiredness = poor work-life balance. AND lack of limited compensations = financial loss = stress | **WORK-LIFE BALANCE**. No energy to socialize = poor mental wellbeing. **PHYCHOSOCIAL HAZARDS**. High demands = long work hours = poor mental wellbeing AND frightening situations = stress = poor mental wellbeing. **TRAFFIC RISKS.** Parking fines and difficulties, bikes stolen = stress = poor mental wellbeing. |
| **Mbare 2024** | ALGORITHMIC MANAGEMENT. Lack of transparency in the functioning of algorithms = Physical strain , exhaustion, stress | INCOME. Income unpredictability = stress | PSYCHOSOCIAL HAZARDS. Work demands (workpace, workload), Work control (Task discretion) = Physical strain , exhaustion, stress . Workplace support (Organisational support) = stress. WORK-LIFE BALANCE = Physical strain , exhaustion, stress. |
| **Morita 2022** | x | x | x |
| **Nguyen-Phuoc 2023** | x | x | PSYCHOSOCIAL HAZARDS. Job demands, job resources = burnout, personal resources (self-efficacy, hope, resilience, and optimism)= decrease the chance of burnout |
| **Nilsen 2023** | x | x | **WORKING HOURS**. Among onsite platform workers. Long working hours = sedentary work. |
| **Reimann 2022** | x | x | **PSYCHOSOCIAL. High control** = less somatic health symptoms. **Job flexibility** (temporal and location) = no association with well-being. |
| **Schlicher 2021** | x | x | **WORKING HOURS**. Less working hours = poorer somatic health. **WORK-LIFE BALANCE**. Long working hours = strain-based work-life conflict (drain energy) = poorer health IF primary motivation for platform work is not monetary |
| **Useche, 2024** | x | x | PSYCHOSOCIAL HAZARDS. Job strain, efford-reward imbalance= fatigue (poor general wellbeing) |
| **Wang 2022** | x | x | x |
| **Wu PF, 2022** | x | x | **PSYCHOSOCIAL HAZARDS**. Social support from colleagues (throught group chats) is positively associated with mental well-being |
| **Wu J, 2022** | x | x | **PYSCHOSOCIAL**: work-related basic psychological need are met = good psychological wellbeing |
| **Yoo, H, 2024** | x | x | x |
| **Zhang, 2022** | **ALGORITHMIC MANAGEMENT**. Workers need to forget about their psychological and physical wellbeing | x | x |
| **Zheng, 2023** | x | x | PSYCHOSOCIAL HAZARDS. Higher work pressure = Higher prevalence of occupational injuries. Weekly orders (demands) = the higher the demands the higher the prevalence of occupational injuries (but with an inverted U shape). SAFETY TRAINING. Reduce occupational injuries |
| **Zong, 2024** | x | x | Traffic risks = injuries |
| **ILO 2021.** | x | **INCOME**. Delivery and taxi platform workers. Insufficient payment = stress. | Increase STRESS for online platform workers: atypical and long working hours, **job insecurity**, poor **work-life balance** (23% reported to be stressed). Increase stress for onsite platform workers: **Traffic risks** (traffic congestion, risk of work-related injury), **Working hours** (Long waiting times, long working hours), **psychosocial hazards** (time pressure to drive quickly, insufficient number of rides, risk of crime) |
| **HUWS, 2017** | **ALGORITHMIC MANAGEMENT**. Deactivation of the account = stress, depression | x | **ERGONOMIC HAZARDS**. Online platform workers. Prolonged sitting= back pain. **TECHNO HAZARDS**. Online platform workers. Exposure to screens= eye-strain. **PHYSICAL HAZARDS**. On-site platform workers. Exposed to iron = burns (injuries). **LONG WORKING HOURS**. On-site platform workers. Long hours = many health hazards (musculoskeletal pain, stomach issues). **WORK-LIFE BALANCE**. Long working hours= no free time = depression. **PSYCHOSOCIAL HAZARDS**. Unpredactibility of tasks = stress |

**Table S5.** **Characteristics of the included studies: general results and study of axes of inequality.**

| **Author, year** | **General results (results related with the health of platform workers that can not be classified to be related to business practices, employment or working conditions)** | **Axes of inequality: do they provide health effects separately by axes of inequality (sex, age, country of birth…)** |
| --- | --- | --- |
| **AbdSamad 2023** | the main motivation for choosing gig work is flexible work hours. The challenges faced by gig workers include the meagre ability to save, and lack of retirement security. No health benefits are present to support the sustainable wellbeing of gig workers. | No |
| **Alacovska 2024** | As the primary sources of their fear and anxiety, they most commonly cited the unreliability, malice, and devious intentions not only on the part of their clients and the platform‐owner (and by extension the algorithm) but also of their globally dispersed fellow‐ platform‐workers. | X |
| **Apouey, 2020** | STRESS and ANXIETY. No association for taxi platform vs. Control group. Protective effects for delivey platform vs. Control group | N/A |
| **Bartel, 2019** | Not having access to taxi-stands = no WC space = dehidration (avoid drinking water to not lose time). Not having easy access to healthy food = unhealthy eating. Exposure to second-hand-smoke = difficult to refuse passengers that smoke (eventhough it's forbidden) due to economic insecurity and risk of bad ratings. | No |
| **Beckman 2021** | Drivers expressed very high levels of concern for exposure and infection (86%–97% were “very concerned” for all scenarios). Only 31% of drivers reported receiving an appropriate mask from the company for which they drive. Stress (assessed via PSS‐4) was significantly higher in drivers who reported having had COVID‐19, and also significantly higher in respondents with lower reported job satisfaction. Informants frequently identified supports such as unemployment benefits and peer outreach among the driver community as ways to ensure that drivers could access available benefits during COVID‐19. | N/A |
| **Berger 2019** | Uber drivers report higher levels of anxiety compared to employees and other self-employed. | No |
| **Boniardi, 2024** | Road accidents were reported by 39% of riders, influenced by type of vehicle, fatigue, and number of daily deliveries. Physical and verbal assaults (12% and 28%, respectively) were reported, as well as health-related issues, particularly musculoskeletal disorders. | They adjust their etimates by these factors. |
| **Caban-Martinez 2020** | 57.1% rated their health as very good/good, over 37% of drivers reported muscle or joint pain, of which the top two most frequently reported areas included the lowback (34.3%) and neck (11.4%) | No |
| **Christie 2019** | Gig work led some couriers to experience impairment caused by fatigue and pressure to violate speed limits and to use their phones whilst driving. Many admitted to having a collision and experiencing near misses daily. In the online survey 42% said they had been involved in a collision where there vehicle had been damaged and 10% said that someone had been injured, usually themselves. Most respondents (75%) said there had been occasions while working when they have had to take action to avoid a collision | No |
| **Christie 2023** | Gig workers were more likely to report being involved in a collision where someone was injured | x |
| **El Bourkadi, 2023** | Uber’s structure imprisons the users’ freedom of negotiation and action, which creates a stressful work environment as managerial algorithmic communication only functions effectively in ideal working conditions, while abnormality is very frequent in a profession like transportation. | x |
| **Glavin, 2021** | Platform workers report greater levels of powerlessness and loneliness compared to non-platform workers. This association is not fully explained by financial strain, suggesting that aspects inherent to platform work contribute to these feelings. Rideshare drivers and online crowdworkers both report statistically significant higher levels of powerlessness and loneliness compared to workers not performing these activities, although the powerlessness/loneliness difference is considerably larger among rideshare drivers. | x |
| **Glavin 2022** | Recent participation in platform work is associated with higher psychological distress. // General (household) financial strain explains part (50%) of the association between dependent platform work and psychological distress - although the distress effect remains after adjusting for psychosocial working conditions. Platform workers experience larger mental health penalies associated with higher financial strain. -----Dependent platform workers ("platform work is their self-reported main job") = report higher levels of psychological distress than all other groups (incl. secondary platform workers) | Age, gender, minority group membership, household composition are added as controls, but no separate health effects were modeled. |
| **Harris 2021** | Approx 11% of the participants reported covid symptoms | No |
| **Hafeez 2023** | digital platforms workers had less stress levels compared to the other groups. | No |
| **Jing Z 2023** | N/A | No |
| **Kim S 2021** | identified five common psychological problems: (a) stress from real-time assessment in public space; (b) exhaustion from working without work-life boundaries; (c) infringement of privacy through exposure of private life; (d) verbal abuse in non-face-to-face relations; and (e) isolation from working alone. | No |
| **Kim M-S 2023** | The prevalence of depressive symptoms is greater among gig workers. | Yes. AGE (much higher proportion of gig workers showed symptoms of depression than general workers when they were 45 years of age or older) |
| **Kurian, 2024** | Higher motivation = higher quality of life, higher challenges = larger is the stress. Quality of life has a significant positive impact on wellbeing while stress has a significant negative impact on wellbeing. | x |
| **Laskaris, Z 2024** | Platform dependency is associated with higher prevalence of occupational injuries, after adjusting for potential confounders such as lenght of employment and working hours. | x |
| **Liu, 2025** | Platform workers showed worse work conditions and health status than general workers. | x |
| **Louzado-Feliciano et al., 2022** | Drivers reported health issues such as stress, changes in sleep behaviour, weight gain and digestion issues. | No |
| **Lu Z, 2023** | Transition into gig work is associated with better mental health compared with those who remained not employed among male. transition into gig work is associated with worse mental health compared with the transition into regular employment | Yes, gender |
| **Mbare 2023** | Possibility to earn money (access to labour market) = enhance mental wellbeing | No |
| **Mbare 2024** | This study established that algorithmic management had direct and indirect intertwined negative psychosocial influence on couriers. These impacts were varied and were related to how algorithmic management was employed by the platforms. We also found that algorithmic management can increase work demands, decrease couriers’ control over their work, and limit workplace support. The study demonstrated that Job Demand-Control-Support model is suitable for analysing stress related to algorithmic management and platform work. | No |
| **Morita 2022** | Gig workers have higher odds of any minor occupational injuries and activity-limiting injuries compared to those not performing gig work. | No, but they adjust by multiple socio-demographic charactersitics |
| **Nguyen-Phuoc 2023** | Job burnout, job resources, and personal demands directly impact risky riding behaviours, in which job burnout was the most significant predictor. | x |
| **Nilsen 2023** | Delivery workers report minor injuries (scratches, bruises and broken ribs) | No |
| **Reimann 2022** | The findings suggest that if crowdworkers use temporal and task flexibility, they experience fewer work-life conflict, fewer somatic health symptoms, and greater life satisfaction. This does not apply to flex-place. The relationship between flexible working and well-being is partly mediated by work-life conflict experiences. | No ,adjusted for only. |
| **Schlicher 2021** | Crowdworkers have worse somatic health than regularly employed workers | AGE. younger platform workers have poorer somatic health than young regularly employed workers |
| **Useche, 2024** | the occupational (riding) crashes of food delivery riders can be largely explained through work-related fatigue, which exerts a full mediation between job settings, stress-related factors and riding safety outcomes | no |
| **Wang 2022** | Gig workers’ worse mental health compared to full and part-time workers were explained by higher levels of loneliness and financial precarity. But their better mental health than unemployed is explained by better financial precarity compared to unemployed. | No, but they adjust by multiple socio-demographic charactersitics |
| **Wu PF, 2022** | The stress buffering effect of social support mainly comes from the riders' familial contact and their online group chat with other workers. Overall, despite the welldocumented precarity and stress in platform work, the riders in our sample appear to be able to mobilize inner and relational resources to achieve a relatively high‐level mental well‐being. | No |
| **Wu J, 2022** | Chinese gig workers pay more attention to whether respect and recognition, and occupational planning are met. This study contributes to the literature of basic psychological need and gig workers’ well-being in the Chinese context. | No |
| **Yoo, H, 2024** | The prevalence of health problems, including musculoskeletal symptoms, general fatigue, and depressive symptoms, in each occupational group was statistically higher than that in the general population after standardization for age and gender. |  |
| **Zhang, 2022** | how various design dimensions of algorithmic management, including information asymmetries and unfair, manipulative incentives, hurt worker well-being. Workers generate designs to address these issues while considering competing interests of the platforms, customers, and themselves, such as information translucency, incentives co-confgured by workers and platforms, worker-centered data-driven insights for well-being, and collective driver data sharing. | They find similar concerns for age groups, gender, background |
| **Zheng, 2023** | Platform dependency: occupational injury rate of those who rely mainly on their platform income is 10.92% higher than those who rely less on platform income. | adjusted for axed of inequality |
| **Zong, 2024** | The results revealed five types of hassles experienced by platform-based food couriers: hassles from food platforms, hassles from on-demand restaurants, hassles during food delivery rides, hassles from customers, and hassles in personal life. | x |
| **ILO 2021.** | Differences between onsite platform work and traditional work. Traditional taxi drivers (2%) report poorer health than platform taxi workers (1%), the same for traditional delivery workers (3%) report poor health than platform delivery workers (1%). Differences across types of online platform work. Competitive programming workers report poorer health than freelancers and microtaskers. Access to labour market. Workers with disabilities have the possibility to access the labour market because of the flexibility to work from home. | Yes. REGION (developed vs developing countries) & SEX (female vs male). Online platform workers in developed countries, females have poorer self-reported health. |
| **HUWS, 2017** | x | No |
